# Effect of salbutamol-based anti-asthmatic medication on surface roughness and color stability of different types of hybrid ceramics

**DOI:** 10.1038/s41405-025-00335-7

**Published:** 2025-05-20

**Authors:** Noha Attia, Waleed Elshahawy, Abeer Atef Younes

**Affiliations:** Department of Fixed Prosthodontics, Faculty of Dentistry, Tanta University, Tanta, Egypt

**Keywords:** Health care, Fixed prosthodontics

## Abstract

**Objective:**

To evaluate the impact of salbutamol sulfate inhalation on the surface roughness and color stability of hybrid ceramics using an inhaler and nebulizer at different doses over three time periods.

**Materials and methods:**

A total of 120 samples of Lava Ultimate (LU), Cerasmart (CS), and Nacera Hybrid (NH) were divided into three equal groups. Each group was subdivided into two subgroups according to the use of inhaler or nebulizer. Each subgroup was subdivided into two divisions according to the minimum and maximum doses of inhalation. The samples were cut rectangular and polished according to the manufacturers’ instructions. They were exposed to salbutamol in acrylic boxes and then kept in artificial saliva for 30, 60, and 90 days which was equivalent to 4, 8, and 12 months. The surface roughness and color change (ΔE) were measured before and after exposure to salbutamol via a non-contact profilometer and a reflective spectrophotometer based on CIE L*a*b* respectively. The data were tabulated and statistically analysed using a three-way ANOVA test and the significance level was set at *P* ≤ 0.05.

**Results:**

Cerasmart recorded the highest statistically non-significant roughness change (1.2298 ± 0.331%, *P* > 0.05). NH had the highest statistically significant ΔE (ΔE = 6.174, *P* ≤ 0.0001). The maximum dose used in three-month exposure period had the highest statistically significant change in roughness and color (*P* ≤ 0.0001). Additionally, the inhaler-treated groups presented significantly higher values than the nebulizer-treated groups for ΔE only (*P* < 0.05). Eventually, Pearson correlation analysis revealed a statistically significant positive direct relationship between roughness and ΔE (*P* = 0.013).

**Conclusions:**

Salbutamol can significantly affect the color of hybrid ceramics while causing a non-significant increase in their surface roughness.

## Introduction

Asthma represents a major chronic respiratory noncommunicable inflammatory condition, marked by recurring episodes of difficulty breathing and wheezing. The intensity and frequency of these episodes can differ widely among individuals [1]. The management of asthma is based on pharmacological therapy involving either anti-inflammatory drugs such as low-dose inhaled corticosteroids (ICSs) or bronchodilators such as short-acting β_2_ agonists (SABAs) or their combination [2]. These therapeutic options can be administered via different routes such as inhalation, orally, or parenterally. The inhaled route has become more popular because it offers two main benefits: it reduces systemic concentrations and the possibility of side effects while optimizing concentrations in the target tissue for the maximum therapeutic effect [3, 4]. One of the typical examples of inhaled drugs is salbutamol sulfate. Salbutamol is a class of β_2_ adrenergic agonist that is the most potent bronchodilator for treating acute asthma attacks [5]. The adverse effects of salbutamol have been a subject of interest as Sivaramakrishnan et al. (2023) reported that it can lead to a reduction in the production of saliva and decrease its pH, which can affect the natural way in which the mouth maintains its chemical balance and increase the risk of dental erosion [6].

Computer-aided design and computer-aided manufacturing (CAD/CAM) materials with a dual network structure of ceramic and polymer are known as ceramic-reinforced polymers or hybrid ceramics. The rationale behind this category of materials was to merge the benefits of polymers, which include reduced antagonist wear and enhanced flexural properties, with those of ceramics, which are characterized by color stability and structural durability [7]. However, these materials have disadvantages such as wear, low fracture strength, and discolouration [8].

According to Labban et al. (2021), hybrid materials exhibit mechanical properties, that imitate the enamel structure. Therefore, these materials could be substitutes for glass ceramics [9]. These materials include various materials with different compositions of resin and polymers such as Lava Ultimate, Cerasmart, and Nacera Hybrid. They are claimed to cover the indications of onlays, inlays, endocrowns, and crowns [10].

The lack of research on how anti-asthmatic drugs influence the color and surface roughness of hybrid ceramics indicates the need for more investigations on this subject. This in vitro study aimed to determine how inhalation of the anti-asthmatic medication “salbutamol sulfate” affected the surface roughness and color stability of different hybrid ceramics (LU, CS, and NH) when inhaler and nebulizer inhalation devices were used with their minimum and maximum doses at three evaluation periods. The null hypothesis was that “Salbutamol sulfate would not influence the color and surface roughness of hybrid ceramics at varying dosages after 30, 60, and 90 days”.

## Materials and methods

### Ethical approval and preparation of samples

The design and procedures of this study were accomplished according to the research guidelines adopted by the Research Ethics Committee at the Faculty of Dentistry, Tanta University, Egypt, with code (FP 07-22 3).

The sample size was estimated using GraphPad StatMat 2.00 software (Graph Pad Inc., Boston, United States). Based on the pilot study, the required sample size came up to 40 in each group which has a 90% power to detect a difference between means of 0.36 with a significance level (alpha) of 0.05 (two-tailed), effect size 0.728 and 95% confidence interval. In 90% (the power) of those experiments, the *P* value will be less than 0.05 (two-tailed) so the results will be deemed “statistically significant”. In the remaining 10% of the experiments, the difference between means will be deemed “not statistically significant”,

$$N={\left(\frac{Z\delta }{{ME}}\right)}^{2}$$

where; (Z) is the Z score, (δ) is the standard deviation and (ME) is the margin of error.

Overall, 120 samples of three different materials were tested, and presented in Table 1. These materials, Lava Ultimate (LU), Cerasmart (CS), and Nacera Hybrid (NH), were divided into three equal groups (*n* = 40). Each group was subdivided into two subgroups based on inhaler or nebulizer usage (*n* = 20). Each subgroup was further subdivided into two divisions based on the minimum and maximum inhalation doses (*n* = 10).

**Table 1:** Materials tested in this study.

Material	Product	Manufacturer	Composition	Shade	Lot / batch number
Resin-nanoceramic CAD/CAM block	Lava Ultimate (LU)	3M ESPE, St. Paul, MN, USA	-Filler: 80% SiO_2_ (20 nm) and ZrO_2_ (4-11 nm) nanoparticle & aggregated ZrO_2_/SiO_2_ cluster -Matrix: 20% BisGMA, UDMA, BisEMA, TEGDMA	A2-HT	N933366
Resin-nanoceramic CAD/CAM block	Cerasmart (CS)	GC Corporation, Tokyo, Japan	-Filler: 71% SiO_2_ (20 nm) and barium glass (300 nm) nanoparticles -Matrix: 29% BisMEPP, UDMA, DMA	A2-HT	2104121
Resin-ceramic CAD/CAM block	Nacera Hybrid (NH)	Doceram Medical Ceramics Gmbh, Dortmund, Germany	-Filler: 50% nano glass -Matrix: 50% polymer matrix	A2	5046268

*BisEMA* ethoxylated bisphenol A dimethacrylate, *BisGMA* bisphenol A glycidil methacrylate, *BisMEPP* Bisphenol A ethoxylate dimethacrylate, *DMA* dimethacrylate, *SiO*_*2*_ Silicon dioxide, *TEGDMA* triethylene glycol dimethacrylate, *UDMA* urethane dimethacrylate, ZrO_2_ zirconium dioxide.

An automatic section low-speed diamond saw with a water cooling system (Isomet 4000; Buehler, Lake Bluff, Illinois, USA) was used to cut the samples from the tested hybrid CAD/CAM blocks into rectangular shapes that measure (14 mm in length, 12 mm in width and 1.5 mm in thickness) at a speed of 2500 rpm [11].

The samples were finished sequentially with wet silicon carbide abrasive papers 600, 800, and 1000 grids with low-speed handpiece (model number: CX235C1-2; COXO Inc, Guangdong, China) at 300 rpm to standardize the surface roughness of all the samples. The polishing protocol was carried out with the manufacturer’s suggested polishing kit for each material, as shown in Table 2. To simulate the clinical intraoral procedures, the finishing and polishing protocol was carried out on the same surface of the samples in one direction with standardized light finger pressure by the same operator. After the manual polishing process, the samples were cleaned in an ultrasonic cleaner (Jiayuanda Tech. Co., Ltd., Shenzhen, China) containing distilled water for 10 min, followed by air drying. The sample thickness was confirmed with a digital caliper (model number: ISZ-1108-150; INSIZE CO., LTD, Jiangsu, China) for standardization. The control (baseline) measurements for the test groups will be determined by measuring the surface roughness and color stability of each sample before exposure to the anti-asthmatic medication.

**Table 2:** Polishing protocol.

Material	Polishing steps
Lava Ultimate (LU)	- EVE Diacomp Plus Twist polishing kit (Diacomp Plus Twist; EVE Ernst Vetter GmbH, Keltern, Germany) in two steps below 10,000 rpm (3000–8000) for 15 s in each step: (1) pink spiral rubber discs (medium-grit) for pre-polishing (2) gray spiral rubber discs (fine-grit) for high shine polishing - Goat hair brush (SHINY S goat brush wheel; ENA HRI, Italy) and felt wheel (SHINY F felt wheel; ENA HRI, Italy) + Diapolisher paste (Diapolisher Paste; Shofu, Kyoto, Japan) at 10,000 rpm for 15 s
Cerasmart (CS)	- Compomaster finishing and polishing kit (Compomaster; Shofu Dental Corp, Kyoto, Japan) at 10,000 rpm for 15 s in each step: (1) Compomaster coarse polishers (yellow polisher) for finishing & basic polishing (2) Compomaster fine polishers (with yellow and white band) for superpolishing - Goat hair brush and felt wheel+ Diapolisher paste at 10,000 rpm for 15 s
Nacera hybrid (NH)	- EVE Diacomp Plus Twist polishing kit in two steps below 10,000 rpm (3000–8000) for 15 s in each step: (1) pink spiral rubber discs (medium-grit) for pre-polishing (2) gray spiral rubber discs (fine-grit) for high shine polishing - Goat hair brush and felt wheel + Diapolisher paste at 10,000 rpm for 15 s

### Sample exposure to anti-asthmatic medication and storage in artificial saliva

**Fig. 1:**
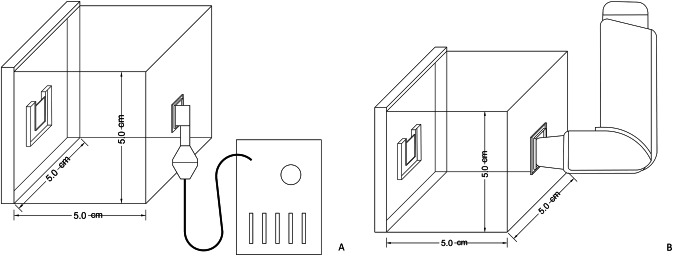
Diagram showing the exposure of the samples to salbutamol sulfate in an acrylic box through. **A** a nebulizer mouthpiece and **B** an inhaler.

Acrylic boxes with dimensions 5 cm in length, 5 cm in width, and 5 cm in height were designed with CorelDRAW X8 software (Corel Corporation, Ottawa, Canada) for the exposure of the prepared samples to salbutamol sulfate so that the drug was concentrated and not spread outside. The interface software then converts these drawings into a series of commands for the laser computer numerical control (CNC) cutting machine. The inner wall center of the acrylic box had a supporting frame to hold the sample vertically to expose the majority of the surface to the medication. The opposite wall of the box had an opening for the inhaler or nebulizer mouthpiece according to their design as illustrated in Fig. 1 [12].

The samples were exposed to two different types of inhalation (nebulizer and inhaler): In the nebulizer subgroup, the samples were exposed to 10 mg and 20 mg of salbutamol sulfate solution (Farcolin respirator solution®; Pharco Pharmaceuticals, Alexandria, Egypt), where undiluted 60 drops (2 ml) of Farcolin solution are equivalent to 10 mg of salbutamol through a nebulizer machine (NE100 Piston Nebulizer; Rossmax Swiss GmbH, Heerbrugg, Switzerland) for 5–10 min daily [13, 14]. In the inhaler subgroup, the samples were subjected to a salbutamol inhaler (Ventolin® Evohaler®; GlaxoSmithKline, London, United Kingdom) at 400 μg and 800 μg daily. The inhaler was agitated until the components were well combined; then, it was held vertically, with its funnel directed at the sample to deliver 100 μg of salbutamol sulfate on each spray [12].

After each salbutamol sulfate exposure, the samples were immersed in 10 ml of artificial saliva in sealed coded glass containers and stored in an incubator (Shaking Incubator “LSI-3016A”; DAIHAN Scientific Co., Ltd, Gangwon-do, Korea) at 37 °C, after which the artificial saliva was replaced every day during the research process. The artificial saliva was prepared with the modified method of Macknight-Hane and Whitford [15]. The previous procedures, including exposure to salbutamol sulfate and immersion in artificial saliva, were applied to every sample every day for 30, 60, and 90 days which is equivalent to 4, 8, and 12 months, respectively [13]. The surface roughness and color change were recorded at the end of each month and entered into the data analysis.

### Surface roughness and color measurement

The surface roughness and color values of all the samples were measured by a non-contact profilometer (NANOVEA Inc, Irvine, United States) and a reflective spectrophotometer (model RM200QC; X-Rite, Neu-Isenburg, Germany), respectively. Three different areas (at the center of the specimen, 1 mm to the right, and 1 mm to the left) of (10 µm × 10 µm) in size were evaluated in each sample to determine the surface roughness value which was averaged to its mean value (Ra) [12]. At a fixed magnification of 90X, photos of the samples were captured using a USB digital microscope (U500X Capture Digital Microscope; Shenzhen Texon Technology Ltd., Guangdong, China) with an integrated camera linked to a compatible personal computer. A three-dimensional representation of the sample surface was generated and examined via WSxM software (version 5 develop 4.1, Nanotec Electrónica S.L., Madrid, Spain), which was used to calculate average of heights expressed in μm so it can be assumed as a reliable indices of surface roughness (Ra) [16].

The color of each sample was measured at the same three measurement points and the average color was recorded. The aperture size was adjusted to 4 mm, and the specimens were exactly aligned with the device. A white background was selected and measurements were made according to the CIE L*a*b* color space relative to the CIE standard illuminant D65. The spectrophotometer was calibrated before each measurement. The color changes (ΔE) of the specimens were evaluated using the following formula:

$${{{\Delta }}{{\rm{E}}}}_{{{\rm{CIELAB}}}}=({\Delta {{\rm{L}}}}^{* 2}+{\Delta {{\rm{a}}}}^{* 2}+{\Delta {{\rm{b}}}}^{* 2})\frac{1}{2}$$

where L* is the lightness coefficient, ranging from black (=0) to white (=100), a* is the shade of redness (positive values) and greenness (negative values), and b* indicates yellowness (positive values) and blueness (negative values) [17].

### Statistical analysis

The data were tabulated and statistically analysed using three-way and multiple factorial ANOVA tests for each tested factor (material group, treatment type, dose, and evaluation time). One-way analysis of variance was used to analyse the data for each factor, and if significant results were found, Tukey’s post-hoc test was used. A Student’s t-test was used to compare paired groups. Pearson correlation was used to identify the relationship between color and roughness change. The results were analysed using Graph Pad InStat (Graph Pad Inc., Boston, United States) software for Windows. A value of *P*<0.05 was regarded as statistically significant.

## Results

### Surface roughness

**Fig. 2:**
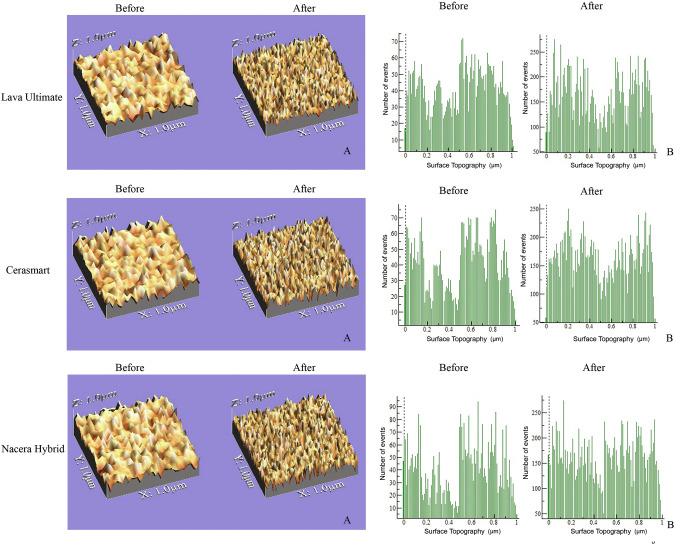
Surface roughness of Lava Ultimate, Cerasmart, and Nacera Hybrid before and after 3 months of salbutamol exposure evaluated through. **A** 3D images of the surface topography converted by computer software and **B** Mathematical histogram.

The mean values and standard deviations of surface roughness (µm) and change percentage (%) for all material groups and treatment types with minimum and maximum doses in the three evaluation periods are summarized in (Tables 3 and 4 respectively). The baseline Ra values for CS (0.2865 ± 0.003 µm) showed an increase to (0.2937 ± 0.002 µm) after three months exposure to maximum dose of inhaler. Among the material groups, CS had the highest roughness change (1.2298%) followed by NH (1.1225%), whereas the lowest value was recorded with LU (1.0741%), indicating a statistically non-significance difference as confirmed by a multifactorial ANOVA test (*P* > 0.05), as shown in (Table 5). The inhaler-treated groups presented a statistically non-significant increase in roughness change (1.2275%) compared with the nebulizer-treated groups (1.057%) (*P* > 0.05), as illustrated in Table 5. The maximum dose-treated groups presented a statistically significant roughness change (1.3186%) compared with the minimum dose-treated groups (0.9656%) (*P* < 0.05), as shown in Table 5. The highest roughness change was recorded after three months (1.6057%), followed by two months (1.123%), whereas the lowest mean value was observed after one month (0.6977%), which was statistically significant (*P* < 0.05), as shown in Table 5. The surface roughness of all the samples in the tested groups was evaluated before and after salbutamol exposure through optical images with a digital microscope, and then converted by computer software into 3D images of the surface topography and mathematical histogram, as shown in Fig. 2.

**Table 3:** Surface roughness (µm) results for all material groups and treatment types with minimum and maximum doses in three evaluation periods.

		Min. dose				Max. dose			
		Inhaler		Nebulizer		Inhaler		Nebulizer	
		Mean	SD	Mean	SD	Mean	SD	Mean	SD
Baseline	LU Gr	0.2896	0.002	0.2900	0.0011	0.286	0.019	0.2900	0.01
	CS Gr	0.289	0.002	0.289	0.002	0.2865	0.003	0.289	0.002
	NH Gr	0.2887	0.006	0.2877	0.002	0.2881	0.001	0.2882	0.002
One month	LU Gr	0.2922	0.005	0.2906	0.0048	0.29	0.001	0.2903	0.001
	CS Gr	0.2908	0.005	0.291	0.005	0.2911	0.003	0.291	0.003
	NH Gr	0.2891	0.002	0.2906	0.003	0.2886	0.003	0.2906	0.002
Two months	LU Gr	0.2925	0.004	0.2923	0.001	0.293	0.002	0.2911	0.004
	CS Gr	0.2909	0.002	0.291	0.001	0.2918	0.002	0.292	0.002
	NH Gr	0.2912	0.003	0.2935	0.003	0.2889	0.024	0.2924	0.002
Three months	LU Gr	0.2929	0.002	0.2931	0.0029	0.294	0.003	0.2919	0.005
	CS Gr	0.2927	0.004	0.293	0.003	0.2937	0.002	0.294	0.001
	NH Gr	0.2912	0.003	0.2936	0.004	0.2926	0.005	0.2946	0.004

*SD* standard deviation.

**Table 4:** Surface roughness change (%) results for all material groups and treatment types with minimum and maximum doses in three evaluation periods.

		Minimum dose				Maximum dose			
		Inhaler		Nebulizer		Inhaler		Nebulizer	
		Mean	SD	Mean	SD	Mean	SD	Mean	SD
One month	LU Gr	0.8978	0.004	0.2069	0.003	1.3986	0.01	0.1034	0.006
	CS Gr	0.6228	0.004	0.692	0.004	1.6056	0.003	0.692	0.003
	NH Gr	0.1386	0.004	1.008	0.003	0.1736	0.002	0.8328	0.002
Two months	LU Gr	1.0014	0.003	0.7931	0.001	2.4476	0.011	0.3793	0.007
	CS Gr	0.6574	0.002	0.692	0.002	1.8499	0.003	1.0381	0.002
	NH Gr	0.866	0.005	2.016	0.003	0.2777	0.013	1.4573	0.002
Three months	LU Gr	1.1395	0.002	1.069	0.002	2.7972	0.011	0.6552	0.008
	CS Gr	1.2803	0.003	1.3841	0.003	2.5131	0.003	1.7301	0.002
	NH Gr	0.866	0.005	2.0507	0.003	1.562	0.003	2.2207	0.003

*SD* standard deviation.

**Table 5:** Roughness change (%) results for all material groups, treatment type, dose, and evaluation time.

		Roughness change (%)		Statistics
		Mean	SD	*P* value
Material group	LU Gr	1.0741	0.282	0.1114 ns
	CS Gr	1.2298	0.331	
	NH Gr	1.1225	0.389	
Treatment type	Inhaler	1.2275	0.464	0.0562 ns
	Nebulizer	1.057	0.505	
Dose	Minimum	0.9656	0.358	0.0002*
	Maximum	1.3186	0.612	
Evaluation time	One month	0.6977	0.137	<0.0001*
	Two months	1.123	0.042	
	Three months	1.6057	0.127	

*SD* standard deviation, * significant (*P* < 0.05), (*P* > 0.05) is non-significant (ns).

### Color change

**Fig. 3:**
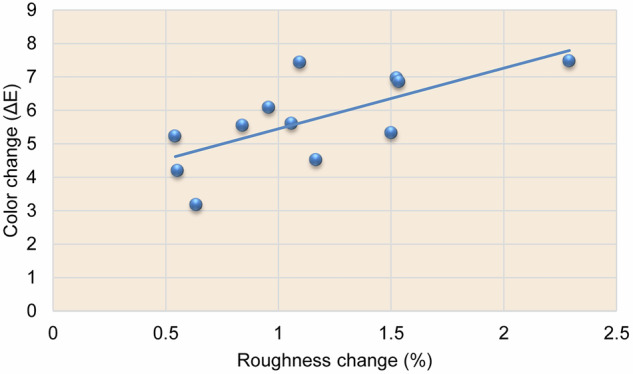
Linear chart of the correlation between total color change and roughness change (%).

The color change (ΔE) mean values and standard deviation results for all material groups with different doses and types of inhalation during the three evaluation months are shown in Table 6. These results can be summarized over the whole period of the study, as ΔE between material groups was statistically significant, where NH (ΔE = 6.174) had the highest ΔE, followed by LU (ΔE = 5.7083), while CS (ΔE = 5.235) showed the lowest results as confirmed by a multifactorial ANOVA test (*P* ≤ 0.0001). Concerning the evaluation time, the three-month exposure period showed statistically significant higher ΔE followed by the two-month period, and the lowest change occurred at the one-month period (*P* ≤ 0.0001). Compared with the inhaler-treated groups, the nebulizer-treated groups showed a significantly lower ΔE (*P* ≤ 0.0001). The maximum dose-treated groups had statistically significant higher ΔE than minimum doses-treated groups (*P* ≤ 0.0001). Eventually, a statistically significant direct relationship was documented between color and roughness change (%) as revealed by the Pearson correlation coefficient (*r* = 0.6898, *P* < 0.05), as shown in Fig. 3.

**Table 6:** Color change results for all material groups related to three evaluation months and type of inhalation used with minimum and maximum doses.

		Minimum dose				Maximum dose				Statistics
		Inhaler		Nebulizer		Inhaler		Nebulizer		*P* value
		Mean	SD	Mean	SD	Mean	SD	Mean	SD	
One month	LU Gr	4.808^A^	0.85	3.9^A^	0.82	5.303^A^	1.45	5.99^A^	1.17	0.0014*
	CS Gr	2.995^B^	0.86	3.099^A^	0.77	6.154^A^	1.33	4.06^B^	0.8	<0.0001
	NH Gr	4.809^A^	2.53	2.539^B^	0.73	5.382^A^	1.42	5.63^A^	2.14	0.0023*
Two months	LU Gr	5.617^A^	1.22	4.45^A^	0.95	6.316^B^	1.46	6.28^A^	1.87	0.0186*
	CS Gr	4.175^B^	0.61	4.529^A^	1.38	6.433^B^	0.61	5.81^A^	1.53	<0.0001*
	NH Gr	6.86^A^	2.27	4.587^A^	0.82	8.166^A^	1.3	6.19^A^	0.86	0.0023*
Three months	LU Gr	6.291^B^	1.63	4.744^A^	1.08	7.121^B^	0.92	7.68^A^	0.95	<0.0001*
	CS Gr	6.423^B^	1.81	6.304^A^	2.95	6.778^B^	0.75	6.06^B^	1.24	0.8577 ns
	NH Gr	9.607^A^	0.95	4.934^A^	1.22	8.537^A^	0.89	6.85^B^	1.63	<0.0001*

Different letters in same column indicating significant between groups (*P* < 0.05) *significant (*P* < 0.05), *ns* non-significant (*P* > 0.05).

## Discussion

Based on the findings of the present study, the null hypothesis was partially rejected, as salbutamol sulfate caused a statistically non-significant increase in the surface roughness of all the tested materials, whereas a statistically significant change in color after exposure to the medication was documented. Salbutamol sulfate was chosen for this in vitro study because it is typically used to treat acute symptoms in asthmatic patients. Also, it can be added to controller medication in patients with chronic persistent asthma. Furthermore, it is the most commonly applied example of an acidic oral medication leading to “medication-induced dental erosion” [18].

In the present study, salbutamol was applied via two delivery devices: a pressurized metered-dose inhaler (pMDI) and a nebulizer. The pMDI has numerous benefits as documented by Geller et al. (2005), owing to its small size, portability, and convenience, with a short administration time but it requires adequate patient coordination to synchronize inhalation with actuation and only 10–20% of the dose deposited in lungs which can lead to adverse effects [19]. Furthermore, Marques et al. (2022) reported that nebulized salbutamol has maximal bronchodilation, do not require hand-breath coordination and easy to use for elderly patients but is high in cost [1].

Concering the dose of salbutamol, there is no exact prescribed dose of β_2_ agonist for the management of asthma, as it is an “as-needed” medication. Therefore, Puspitasari et al. (2021) reported that in case of a metered-dose inhaler, the dose can vary from 400 μg to 800 μg, which is the maximum dose inhaled daily [12]. The nebulizer dose can range from 10 mg to 20 mg, which is the maximum dose to be administered daily, in agreement with the findings of Paravallika and his colleagues [13]. The duration of the treatment in the current study was adjusted according to the guidelines of “The National Asthma Education and Prevention Program (NAEPP) Expert Panel Report-3 (EPR-3)”; thus, exposure to salbutamol for 30, 60, and 90 days corresponds clinically for 4, 8, and 12 months of exposure, respectively [18, 20].

In the present study, the surface roughness and color affected by anti-asthmatic medication may be attributed to its composition, which includes many active ingredients such as citric acid, sulfuric acid, sucrose, sodium benzoate and ethyl alcohol. Therefore, sulfuric acid and citric acid play essential roles, causing the pH of salbutamol to equal 3.64, which is low enough to be a potent erosive agent. Additionally, sulfuric acid, being a potent acid as opposed to the citric acid included in Ventolin, has a reduced pKa value and undergoes a reduction to sulfur dioxide owing to the presence of alcohol, thus reducing its erosiveness, as reported by Candan et al. (2021)[21].

A rough surface may cause several problems such as plaque accumulation, discoloration, and secondary caries due to bacterial adhesion. Also, it can cause antagonist tooth abrasion and decreased fracture resistance of the material due to surface microcracks [22]. Siddanna et al. (2021) stated that, the critical threshold surface roughness for bacterial adhesion has been reported to be 0.2 μm so creating a surface finish smoother than this threshold becomes a priority [23].

Regarding the surface roughness results of the present study, Backer et al. (2017) attributed this increase as being equal to the baseline to the degradation of the silane coupling agent, which bound particles to the polymer matrix, causing surface particles to leach after acid treatment [10]. With respect to the roughness values of the tested materials, the CS surface roughness significantly increased compared with LU, as reported by Çakmak et al. (2021), because of the differences in microstructure, filler content, and filler particle size [24]. In contrast with these results, Egilmez et al. (2018) reported that acid exposure did not affect the roughness of either Lava Ultimate or Cerasmart. This could be attributed to the use of different acid solutions (0.06 M HCl) for a short period of time (24 h) [8].

According to Sagsoz et al. (2019), degradation of dental ceramics when exposed to acidic substances can be attributed to direct attack towards the polymeric matrix, leaching of filler particles, and selective releasing of alkaline ions, which are less stable in the glassy phase than in the crystalline phase. This degradation causes a decrease in hardness value, affect the clinical success and the longevity of ceramic restorations [25].

In the present study, the value of ΔE for the three hybrid materials was considered clinically unacceptable, according to a report by Cruz et al. (2020), who reported that ΔE value less than 1 was undetectable with the naked eye, whereas a color variation of 3.3 > ΔE > 1, which could be perceived by an experienced operator, was clinically acceptable. In contrast, values of ΔE ≥ 3.3 would be identified by an untrained individual, making it clinically unacceptable [26]. Pravallika et al. (2021) explained this significant discolouration because of the drug active ingredient, which contains a (C_13_H_21_NO_3_)_2_・H_2_SO_4_ sulfate group that probably affected the hybrid ceramic surfaces by creating a pellicle matrix, providing an acidic environment [13].

In the present study, the higher ΔE in LU than in CS can be explained by Stamenković et al. (2021), who reported that CS is a Bis-GMA-free material, whereas LU involves 20% Bis-GMA, which is highly hydrophilic due to its hydroxyl side groups. Therefore, it exhibits superior color stainability [27]. In contrast, Labban et al. (2021) did not observe a significant color change for hybrid ceramics in citric acid storage media, irrespective of surface treatment [9].

In terms of the method of drug administration, the inhaler-treated groups presented higher surface roughness and color changes, as explained by Candan et al. (2021), because of the presence of citric acid in ventolin, which can erode the surface of the dental structure [21]. The maximum dose-treated groups recorded higher roughness and ΔE results than those reported by Puspitasari et al. (2021) due to the acidic conditions, which caused the resin to release monomers in a shorter time [12].

According to Pravallika et al. (2021), the evaluation period in this study was confirmed by their findings that the roughness and color measurements increased from one month to three months for three different resins when subjected to a salbutamol-based nebulizer [13]. In contrast to these results, Sagsoz et al. (2016) reported no significant variation in ΔE values among different immersion times [28]. The results of the Pearson correlation between surface roughness and color were supported by Ayaz et al. (2014), who reported that restorations with superior surface roughness exhibit an increased susceptibility to staining. The texture of the surface influences the color of the restoration, with smooth surfaces reflecting more light than rough surfaces [29].

The limitations of this study include that there are differences between the clinical environment & the in vitro environment such as the amount of saliva, the microbes present in the oral cavity, and frequency of tooth brushing, which probably affect the outcomes. Ceramic restorations are subjected to various thermal, physical and chemical challenges when placed in the oral cavity, which are difficult to reproduce entirely under laboratory conditions.

Another critical concern is the potential ion interactions and leaching upon corrosive attack, which can be explained in future research. Also, this study was performed on flat-shaped samples which are not similar to the actual restorations that may have anatomical features, including grooves and pits which may complicate the polishing procedure and show different degrees of discoloration on the same tooth, unlike the flat surface of our samples, which had the same degree of discoloration on the entire surface.

## Conclusions

Based on the results of this study, the surface roughness of all the tested materials statistically non-significantly increased, while the color significantly increased for the three hybrid materials after exposure to salbutamol sulfate via either the inhaler or nebulizer. Compared with the other tested hybrid materials, Nacera Hybrid showed the highest significant discolouration because of the chemical effect of salbutamol.

Asthmatic patients should adhere to sufficient mouth rinses with a neutral pH or basic mouth rinses immediately after using the salbutamol inhaler to counteract their acidic pH with regular tooth brushing. Further clinical studies are recommended for this topic on asthmatic patients.

## Data Availability

All data supporting the findings of this study are available within the paper and its supplementary information.
